# Genome-wide association study identifies the genetic basis of key agronomic traits in 207 sugar beet accessions

**DOI:** 10.1093/hr/uhae230

**Published:** 2024-08-12

**Authors:** Sufang Wang, Zhiyong Yue, Chao Yu, Ruili Wang, Yang Sui, Yaguang Hou, Ying Zhao, Lingling Zhao, Chunmei Chen, Zhimin Yang, Ke Shao

**Affiliations:** Inner Mongolia Academy of Science and Technology, Hohhot , Inner Mongolia, 010000, China; School of Life Sciences, Northwestern Polytechnical University, Xi’an 710072, China; College of Medicine, Xi’an International University, Xi’an 710077, China; Inner Mongolia Academy of Science and Technology, Hohhot , Inner Mongolia, 010000, China; Inner Mongolia Academy of Science and Technology, Hohhot , Inner Mongolia, 010000, China; Inner Mongolia Academy of Science and Technology, Hohhot , Inner Mongolia, 010000, China; Inner Mongolia Academy of Science and Technology, Hohhot , Inner Mongolia, 010000, China; Inner Mongolia Academy of Science and Technology, Hohhot , Inner Mongolia, 010000, China; Inner Mongolia Academy of Science and Technology, Hohhot , Inner Mongolia, 010000, China; Inner Mongolia Academy of Science and Technology, Hohhot , Inner Mongolia, 010000, China; Inner Mongolia Academy of Science and Technology, Hohhot , Inner Mongolia, 010000, China; Inner Mongolia Academy of Science and Technology, Hohhot , Inner Mongolia, 010000, China

## Abstract

Sugar beet (Beta vulgaris) has emerged as one of the two primary crops, alongside sugarcane, for global sugar production. Comprehensively understanding sucrose synthesis, transport, and accumulation in sugar beet holds great significance for enhancing sugar production. In this study, we collected a diverse set of 269 sugar beet accessions worldwide and measured 12 phenotypes, comprising biomass, soluble sugar content, and 10 taproot-related traits. We re-sequenced 207 accessions to explore genetic diversity and population structure. Then we employed a genome-wide association study (GWAS) and RNA-seq to identify single-nucleotide polymorphisms and genes associated with natural phenotypic variations. Our findings revealed a panel of genes potentially regulating biomass and sugar accumulation, notably the dual-role gene* UDP-glucose 4-epimerase*, which genetically balances sugar accumulation and cell wall synthesis. In summary, this study provides a foundation for molecular breeding in sugar beet.

## Introduction

Sugar beet (Beta vulgaris), a biennial plant in the Chenopodiaceae family, originated along the Mediterranean coast of Europe. Despite its long-term agricultural usage, since last century it has become the second-largest sugar crop worldwide, following sugarcane, with its extensive industrial use for global sugar production [1–3]. Currently, Europe, including Russia, is the largest sugar beet cultivation region, with Germany and the Netherlands leading in breeding efforts. North America and Asia also have significant sugar beet cultivation [4]. Over the past century, advancements in breeding techniques have increased sugar content from 4% to 20%, enhancing sugar production and reducing industry costs. Therefore, developing high-yield sugar beet varieties is crucial to ensure a safe, healthy, and nutritious food supply for the growing global population.

Sucrose, the primary sweetener in human food, is predominantly found in sugar beet. Over the past decade, global sugar consumption has risen from 152 million tons to 173.95 million tons, at an average annual growth rate of 1.76% [5]. Despite the rapid increase in total sugar beet yields, production has reached a bottleneck. To overcome this limitation, selecting and breeding sugar beet accessions with high yields and sugar content is an effective approach. The recent sequencing and assembly of the sugar beet genome have provided valuable genetic information [6, 7], facilitating several studies on sugar metabolism using next-generation sequencing technology [8, 9]. However, a comprehensive understanding of sucrose synthesis, transport, and accumulation in sugar beet remains to be elucidated.

Genome-wide association study (GWAS) has emerged as a powerful tool for identifying single-nucleotide polymorphisms (SNPs) and genes responsible for quantitative trait loci (QTL) under natural variation [10]. GWAS has been successfully applied in various crops, including rice [11–14], foxtail millet [15], soybean [16], cotton [17, 18], quinoa [19], chickpea [20], common bean [21], and maize [22], demonstrating its importance in revealing the genetic basis for plant breeding research [23]. Furthermore, GWAS has been employed to identify genes responsible for sugar accumulation in fruits such as watermelon [24, 25], pear [26], and peach [27].

In this study, we gathered a diverse collection of 269 sugar beet accessions worldwide and measured 12 phenotypes, comprising biomass, soluble sugar content, and 10 taproot-related traits assessed using a three-dimensional point cloud approach as previously described [28]. Then we re-sequenced 207 sugar beet accessions to explore genetic diversity and domestication history. By employing GWAS and RNA-seq, we aimed to identify SNPs and genes associated with the natural variation of these phenotypes, ultimately seeking to determine factors governing sugar content at the genetic level. This comprehensive analysis may lay the foundation for breaking the ceiling of unit sugar yield in molecular breeding.

## Results

### Genome variation map of 269 sugar beet accessions

**Figure 1:**
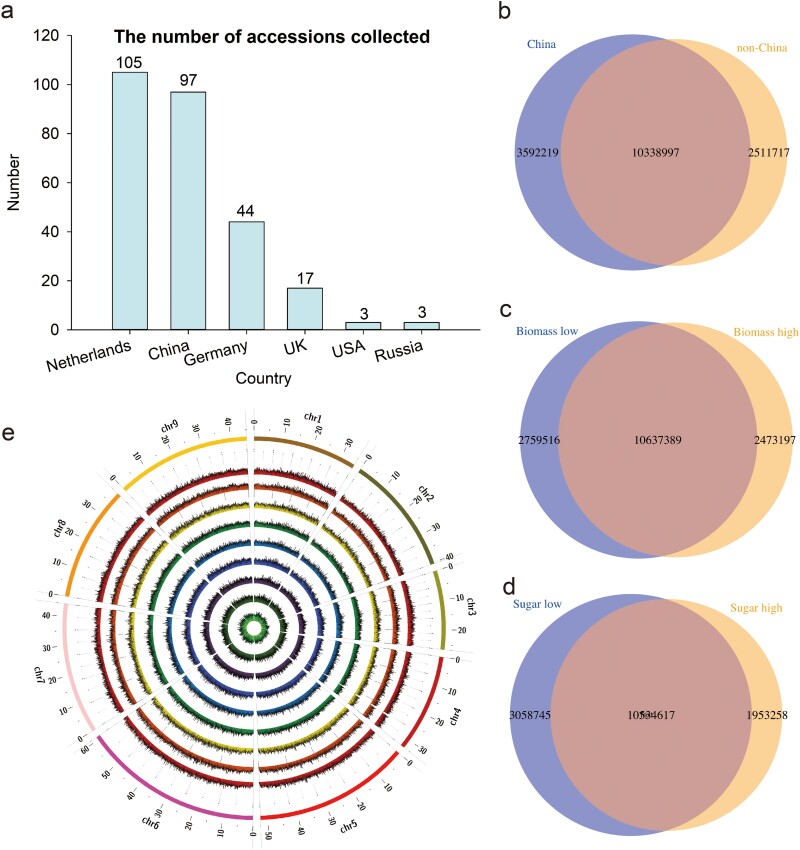
Geographic distribution and genome-wide variations of world-wide sugar beet collection. **a** Bar graph of geographic distribution across 269 sugar beet accessions. **b** Venn diagram representing the numbers of shared and unique SNPs between China and non-China groups. **c** Venn diagram representing the numbers of shared and unique SNPs between high biomass and low biomass groups. **d** Venn diagram representing the numbers of shared and unique SNPs between high sugar content and low sugar content groups. **e** Circos plot of SNP distribution in each chromosome among different groups. Inner circle to outer circle: low sugar content group, middle sugar content group, high sugar content group, low biomass group, middle biomass group, high biomass group, non-China group, China group, and all SNPs. The outermost circle shows the chromosomes (chr1 to 9), indicated by different colors.

A total number of 269 diverse sugar beet accessions were collected worldwide, with 105 from the Netherlands, 97 from China, 44 from Germany, 17 from the UK, 3 from the USA, and 3 from Russia (Fig. 1a, Supplementary Data Table S1). This extensive collection captures the majority of global phenotypic diversity. After sowing seeds and harvesting taproots (with some accessions failing to grow), 216 samples were successfully grown and obtained. Next-generation sequencing technology was used to sequence the sugar beet genomes, resulting in 207 high-quality genomes after quality control filtering, with 9 samples failing. On average, these genomes generated 9 126 583 507 bp of sequence and 15× depth per sample (Supplementary Data Table S2). Following the cleaning of paired-end reads, alignment to the reference genome (version RefBeet-1.2.2) achieved an average mapping rate of 96% per sample (Supplementary Data Table S2).

A total number of 14 615 434 SNPs and 2 978 396 insertions and deletions (indels) were identified across 207 accessions (Table 1), averaging 37.1 SNPs and 6.7 indels per kilobase. Gene regions contained 23% of SNPs and 25% of indels. To investigate genetic differences within this comprehensive set, we categorized accessions into different groups, including three biomass groups (high, middle, and low), three soluble sugar content groups (high, middle, and low), and two regional groups (China and non-China) (Supplementary Data Table S3). Low biomass and low sugar content groups exhibited higher numbers of SNPs and indels compared with their respective high and middle groups (Table 1). Despite having nearly twice the number of accessions in the non-China group, the China group displayed a higher abundance of SNPs and indels (Table 1).

**Table 1:** Genome-wide variations identified in 207 sugar beet accessions.

**Group**	**Number of SNPs**					**Number of indels**				
	**Total**	**Intron**	**Intergenic**	**Exon**	**Others**	**Total**	**Intron**	**Intergenic**	**Exon**	**Others**
All (207)^a^	14 615 434	2 761 039	9 412 647	668 259	1 773 489	2 978 396	699 465	1 732 339	38 501	508 091
China (73)	13 931 216	2 686 701	8 890 797	642 683	1 711 035	2 815 067	671 898	1 620 601	36 724	485 844
Non-China (134)	12 850 714	2 416 356	8 293 671	583 253	1 557 434	2 610 376	611 553	1 520 358	33 612	444 853
High sugar [48]	12 487 875	2 405 457	7 968 155	573 608	1 540 655	2 512 025	599 020	1 444 538	32 535	435 932
Middle sugar (73)	12 845 541	2 470 649	8 190 207	594 996	1 589 689	2 594 878	616 954	1 493 800	33 682	450 442
Low sugar (86)	13 593 362	2 608 715	8 699 098	624 592	1 660 957	2 747 219	654 281	1 585 071	35 489	472 378
High biomass (67)	13 110 586	2 547 091	8 339 049	609 717	1 614 729	2 648 483	635 404	1 520 097	34 431	458 551
Middle biomass (68)	12 398 197	2 373 140	7 931 817	568 695	1 524 545	2 506 874	594 843	1 446 949	32 328	432 754
Low biomass (72)	13 396 905	2 558 114	8 575 999	614 749	1 648 043	2 699 918	640 086	1 558 648	34 993	466 191

a Number of accessions in each group.

To improve the observation of SNP diversity between different groups, we identified the common and unique SNPs. A substantial number of SNPs were shared, with 10 338 997 SNPs being common between the China and non-China groups, 10 637 389 SNPs between the high and low biomass groups, and 10 534 617 SNPs between the high sugar and low sugar groups (Fig. 1b–d). Next, we investigated the genome-wide SNP density across the nine chromosomes, revealing variable distribution, with chromosome 3 having the fewest SNPs and chromosome 6 the highest abundance (Fig. 1e). Overall, this comprehensive dataset of genomic variation serves as a valuable resource for sugar beet breeding and commercial applications.

### Population structure and phylogeny

**Figure 2:**
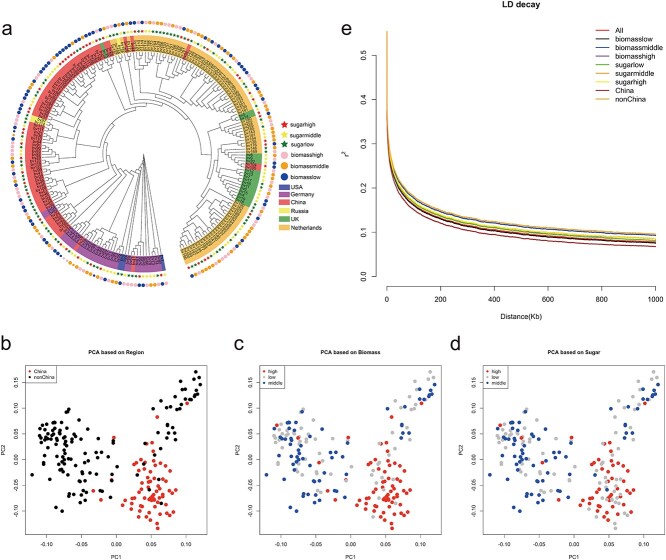
Phylogenetic and population analysis of 207 sugar beet accessions. **a** Neighbor-joining phylogenetic tree of 207 sugar beet accessions. **b**–**d** PCA annotated by region groups, biomass groups and sugar groups. **e** Genome-wide LD decay pattern among different groups.

Phylogenetic analysis of 207 sugar beet accessions using a subset of 4 436 506 SNPs [minor allele frequency (MAF) >0.1, no missing data] revealed two distinct clades with strong geographic separation (Fig. 2a). The first clade comprised accessions from China and Germany, while the second clade comprised accessions from the UK and the Netherlands, reflecting sugar beet breeding history. The reference genome we used was from Germany (KWS2320 DH, version RefBeet-1.2.2), which was assembled by the Max Planck Institute for Molecular Genetics in Germany. According to our phylogenetic analysis, we found that accessions from China and Germany were in one branch, which may be caused by cooperation between China and Germany. Back in the 1990s, Chinese scientists cooperated with Germany and exchanged numerous sugar beet varieties, leading to significant improvements in yield and disease resistance. According to our phylogenetic analysis, we could conclude that Chinese accessions are closer to German accessions and exclude the possibility that the greater number of SNPs identified in the China group compared with the non-China group is due to the reference genome used (Table 1). We also labeled the three biomass groups and three sugar content groups in the phylogenetic tree (Fig. 2a). However, no clear branch pattern can be observed based on phenotypical differences in the phylogenetic tree.

Principal component analysis (PCA) of 207 accessions (using the same number of SNPs for the phylogenetic tree) revealed a distinct separation between the China and non-China groups, indicating a strong geographical distribution pattern (Fig. 2b). However, when we labeled these 207 accessions according to their phenotypical groups in the PCA results, there was no clear pattern (Fig. 2c and d). In addition, compared with non-China accessions, the number of high biomass and high sugar content accessions in the China group was higher than in other groups (Fig. 2b–d), indicating that Chinese scientists have aimed to breed high biomass as well as high sugar content varieties since the last century.

The average nucleotide diversity (π) value for the 207 accessions was 8.6 × 10^−3^. Upon estimating diversity across different groups, both high and low biomass groups exhibited the same nucleotide diversity (8.7 × 10^−3^), while the middle biomass group had a 3.4% lower nucleotide diversity (8.4 × 10^−3^). Notably, the China group had a 4.5% higher nucleotide diversity (8.8 × 10^−3^) compared with the non-China group (8.4 × 10^−3^). As for the high, low, and middle sugar groups, their nucleotide diversity values were 8.7 × 10^−3^, 8.6 × 10^−3^, and 8.5 × 10^−3^, respectively.

To calculate linkage disequilibrium (LD), we determined *r*^2^ between SNP pairs among all groups (three biomass groups, three sugar content groups, two regional groups, and all the accessions in one group). The LD decayed to half of its maximum value (*r*^2^ = 0.3) at a physical distance around 501 kb (Fig. 2e) among all the groups. Due to the heterozygosity of sugar beets, this leads to a fast decay of LD. Upon measuring genome-wide fixation index (*F*_ST_) values based on SNPs across different groups, we identified 71 197 (region group), 1435 (biomass group), and 1894 (sugar group) genomic regions with significant genetic divergence (*F*_ST_ value ≥0.3), which showed greater differentiation in region groups (China and non-China) than in biomass and sugar groups (Supplementary Data Fig. S1). This may indicate a distant evolutionary relationship according to geography (China and non-China). Overall, the examination of population structure and phylogeny uncovers the evolutionary history of sugar beet breeding.

### Phenotype collection and analysis

We collected 12 phenotypic traits from a worldwide collection, comprising two agronomic traits (biomass and soluble sugar content) and 10 taproot traits. The 10 taproot traits were calculated using a three-dimensional point cloud approach as previously described [28], comprising compactness, convex angle, convex index, convex hull volume, maximum diameter, root head ratio, root tail ratio, root taper index, root length, and top projection area. In total, we collected data for 12 traits with replicates (Table 2), but due to germination and environmental factors only 188 accessions were finally obtained (Supplementary Data Table S4). The biomass ranged from 154 to 5728 g (average 1240 g) and soluble sugar content varied between 6.88° and 20.4° (average 16.4°).

**Table 2:** Summary of 12 phenotypical traits.

**Trait**	**Minimum**	**Maximum**	**Median**	**Mean**	**Fold (max/min)**	**Heritability**
Biomass (g)	154	5728	1139.5	1240	37.19	0.91
Sugar (°)	6.88	20.4	16.84	16.43	2.97	0.95
Compactness	0.31	0.86	0.5	0.53	2.77	0.92
Convex angle	0.01	0.7	0.31	0.32	70.00	0.52
Convex index	0.01	0.33	0.16	0.17	33.00	0.51
Convex hull volume	160.87	5098.83	1151.87	1364	31.70	0.91
Maximum diameter	6.13	21.28	11.73	12.22	3.47	0.92
Root/head ratio	0.5	0.99	0.91	0.9	1.98	0.77
Root/tail ratio	0.06	0.64	0.35	0.34	10.67	0.59
Root taper index	0.46	0.97	0.67	0.67	2.11	0.71
Root length	12.95	35.15	22.55	23.21	2.71	0.88
Top projection area	24.99	308.68	92.15	104.12	12.35	0.92

We calculated broad-sense heritability for all traits, with some showing high heritability (>0.9) such as biomass, soluble sugar content, compactness, maximum diameter, and top projection area. In contrast, other traits exhibited low heritability, such as convex angle and convex index (Table 2). This wide range of natural variation and high broad-sense heritability established a foundation for a GWAS.

To better understand the relationship between taproot traits and agronomic traits (biomass and soluble sugar content), we performed a pairwise correlation analysis. Biomass was negatively correlated with soluble sugar content but positively correlated with convex hull volume, maximum diameter, and top projection area (Supplementary Data Fig. S2).

### Genome-wide association study for 12 traits

To identify loci and alleles in 188 accessions, we conducted a GWAS using the efficient mixed model association (EMMA) method. We removed SNPs with a minor allele frequency <0.05, resulting in 4.4 million SNPs for association mapping. The threshold was established at a *P*-value ≤10^−5^, resulting in 143 SNPs identified for biomass and 70 SNPs for soluble sugar content (Supplementary Data Table S5). Then we plotted the *P*-value for each SNP across the whole genome for all 12 traits (Supplementary Data Fig. S3). Notably, the SNPs identified for maximum diameter were also associated with top projection area, suggesting similar genetic control for these two traits. This finding aligns with our previous pairwise correlation analysis results.

Genes within a 20-kb region around significant SNPs were identified due to linkage disequilibrium. The number of genes identified from SNPs for the 12 traits was summarized (Supplementary Data Table S5). Because biomass and soluble sugar content are the most important agronomic traits, we prioritized analyzing genes associated with these two traits. We listed genes identified from each significant SNP for these two traits (Supplementary Data Table S6), including the chromosome, gene ID, and the start and end positions for each gene. Some genes were identified multiple times, and therefore there were 122 unique genes related to biomass and 67 unique genes related to soluble sugar content (Supplementary Data Table S6).

Upon examining whether the significant SNPs altered the amino acid sequences of these 189 genes related to these two traits, we found only two genes with such changes. Since the majority of SNPs were not located within coding regions, this suggests that SNPs may primarily influence gene expression rather than protein activity, leading us to focus on gene expression levels in subsequent analyses. Among these 189 candidate genes, we identified three potential genes (gene ID 104892143, 104890085, and 104889823) that may balance the synthesis between biomass and sugar. These genes are *UDP-glucose 4-epimerase* (ID 104892143), *hydroxyproline O-arabinosyltransferase* (ID 104890085), and *probable pectinesterase* (ID 104889823).

Utilizing three high sugar natural accessions and three high biomass natural accessions as qRT–PCR templates, we designed primers and to obtain the expression levels of these three genes , which exhibited significant differences among accessions (Supplementary Data Fig. S4). *UDP-glucose 4-epimerase* and *hydroxyproline O-arabinosyltransferase* showed significantly higher expression in high biomass accessions, whereas *probable pectinesterase* expression was higher in high sugar accessions (Supplementary Data Fig. S4). This indicated that genes identified from GWAS were differentially expressed and might be targets for molecular breeding in sugar beet.

### Pathways and genes contributing to sugar distribution

**Figure 3:**
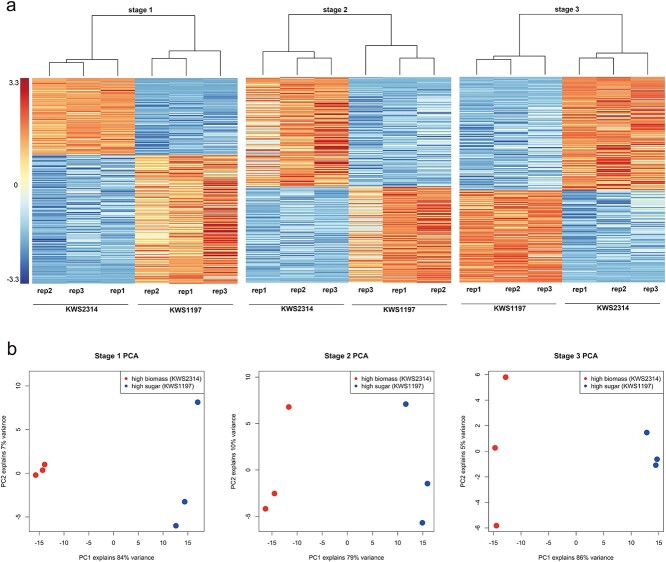
Cluster analysis and PCA using DEGs identified at three stages in RNA-seq experiment. DEGs were defined as log_2_FC ≥ ±1 and FDR ≤ 0.05. Stage 1 is the early developmental stage (foliage rapid growth) with 896 DEGs, stage 2 is the middle developmental stage (root and sugar growth) with 977 DEGs, and stage 3 is the late developmental stage (sugar accumulation) with 617 DEGs.

In order to obtain gene expression at a large scale, two commercial sugar beet accessions, KWS2314 and KWS1197, were selected for an RNA-seq experiment, the former being a high biomass accession and the latter having high sugar content. Samples were collected at three developmental stages: foliage rapid growth (early), root and sugar growth (middle), and sugar accumulation (late). Three replicates were collected per accession for each stage, resulting in a total of 18 samples sequenced using next-generation sequencing technology (Supplementary Data Table S7). Paired-end reads were cleaned and aligned to the reference genome (RefBeet-1.2.2), achieving an average mapping rate of 92% per sample (Supplementary Data Table S7). Differentially expressed genes (DEGs) were identified using DESeq2 with a threshold of log_2_FC ≥ ±1 and FDR ≤ 0.05, yielding 896, 977, and 617 genes for the three stages, respectively (Fig. 3, Supplementary Data Table S8). From the heat maps, these DEGs can separate high biomass and high sugar groups into two clusters, indicating the accuracy of the DEGs (Fig. 3a). PCA using DEGs also showed a clear two-cluster pattern at the three developmental stages. More importantly, principal component 1 can explain 84, 79, and 86% of the variance for the three stages, respectively (Fig. 3b). KEGG pathway analysis was then performed using these DEGs, revealing significant pathways related to sugar metabolism, such as glycolysis, galactose metabolism, and photosynthesis (Supplementary Data Fig. S5).

**Figure 4:**
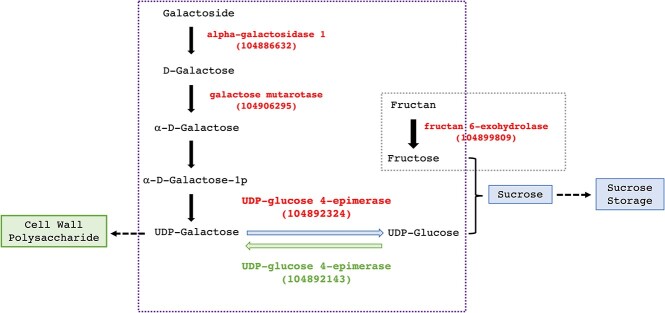
Proposed model of UDP-glucose 4-epimerase regulating sugar distribution. Genes in red font are upregulated genes in high sugar accessions identified from RNA-seq. The gene in green font is the upregulated one in high biomass accessions identified from GWAS and validated by qRT–PCR. During the early growth stage of sugar beet, *UDP-glucose 4-epimerase* (transcript 104892324) showed high expression, resulting in increased glucose production. Concurrently, high fructose hydrolase expression generated higher fructose levels, which, together with glucose, promoted sucrose synthesis. At the late developmental stage, the transcript (ID 104892143) showed high expression and facilitated the conversion of UDP-glucose into UDP-galactose, providing raw materials for cell wall polysaccharide synthesis. These two enzymes work as switches that dynamically regulate sugar distribution.

Interestingly, we identified another gene (ID 104892324) also encoding UDP-glucose 4-epimerase through RNA-seq, which exhibits distinct expression patterns compared with the one (ID 104892143) identified from GWAS. *UDP-glucose 4-epimerase* is responsible for catalyzing the interconversion of UDP-glucose and UDP-galactose. This finding suggests that UDP-glucose 4-epimerases may play a crucial role in regulating the flow direction of hexoses between cell wall synthesis and sucrose storage (Fig. 4). To be specific, during the early growth stage of sugar beet (stage 1 in RNA-seq analysis), *UDP-glucose 4-epimerases* (ID 104892324) exhibited high expression in high sugar accessions, resulting in a higher glucose supply (Supplementary Data Table S8). Concurrently, the elevated expression of fructose hydrolase led to increased fructose levels, which, along with glucose, promoted higher sucrose synthesis in high sugar accessions. However, during the late developmental stage, the other *UDP-glucose 4-epimerase* (ID 104892143) showed higher expression in high biomass accessions, which was identified from GWAS and validated by qRT–PCR (Supplementary Data Fig. S4). This enzyme potentially leads to the acceleration of conversion from UDP-glucose to UDP-galactose, providing more raw materials for cell wall polysaccharide synthesis.

Our phenotype correlation analysis among 188 natural accessions showed an inverse relationship between biomass and sugar content (Supplementary Data Fig. S2). Therefore, the direction of sugar flow is crucial in determining whether accessions are high in biomass or high in soluble sugar content. The different expression patterns of the two UDP-glucose-4-epimerase genes led to a distinct sugar flow direction, suggesting that UDP-glucose 4-epimerases have a dual role in regulating sugar distribution between cell wall synthesis and sucrose storage (Fig. 4).

## Discussion

In this study, we originally collected 269 sugar beet accessions worldwide. After accounting for germination and environmental factors, only 216 accessions were sampled for sequencing. Unfortunately, nine samples did not pass the sequencing quality control. As a result, 207 samples were finally sequenced and used for genotype analysis. By the time we harvested accessions in the field for phenotype measurement, only 188 accessions were finally obtained. A GWAS and RNA-seq analysis revealed genes participating in sugar metabolism pathways. We further identified UDP glucose-4-epimerase as a crucial factor regulating sugar distribution and, consequently, biomass.

The soluble sugar content is a very important agronomic trait, especially in the selection process of sugar beets. It has been found that overexpression of sucrose synthesis in sugarcane can effectively increase sucrose content, indicating that increasing the expression level of genes involved in soluble sugar synthesis is one of the effective approaches to the promotion of soluble sugar accumulation [29]. The transcriptome and metabolome data from two different sugarcane varieties showed that genes and metabolites involved in starch and sucrose metabolism pathways had a significant impact on sucrose accumulation [30]. In addition to increasing the synthesis of sucrose, the rapid transport of soluble sugar is also an important aspect. The TST2.1 protein in sugar beets was responsible for sucrose absorption in the main root vacuoles [31], and SUT1 in sugar beets was responsible for transporting sucrose to the phloem, thereby transporting sucrose to the storage tissue of the main root of sugar beets [32]. Another two glucose transporter proteins, PMT5a and STP13, were also identified recently in the root system of sugar beets [33].

In addition to sugar crops, the mechanism of sugar accumulation in fruit has also been extensively studied; especially, SNPs related to soluble sugar content have been identified in various fruits and crops. A GWAS study on 278 soybean accessions discovered 84 SNPs from 17 genes significantly correlated with soluble sugar content [34]. Furthermore, GWAS on Japanese pear found 56 SNPs and a tandem repeat of *early responsive to dehydration* (*ERD6*)-like sugar transporter genes that may be closely related to soluble sugar content [35]. Thirty-seven SNPs associated with soluble sugar content in citron watermelon were also uncovered recently [36]. These studies have laid the foundation for searching the functional genes regulating soluble sugar content in fruits and crops.

A primary objective in sugar beet breeding is to enhance both sugar content and biomass. However, our study revealed a negative correlation between biomass and soluble sugar content in sugar beet (Supplementary Data Fig. S2), presenting a challenge that requires balancing these two traits. By analyzing GWAS and RNA-seq data, we identified a dual-role gene, *UDP-glucose-4-epimerase*, which may balance cell wall biosynthesis and sucrose storage. At the early developmental stage, this transcript (ID 104892324) is highly expressed, promoting UDP-glucose synthesis and resulting in sucrose accumulation. Conversely, at the late developmental stage, the other transcript (ID 104892143) is highly expressed, leading to increased cell wall synthesis. This enzyme, previously reported in Arabidopsis thaliana, exhibits five isoforms with distinct expression patterns. Two isoforms are primarily involved in carbohydrate catabolism, while the remaining three are more related to carbohydrate biosynthesis [37]. In summary, we showed that these enzymes work as switches that dynamically regulate sugar distribution.

To enhance soluble sugar content, four key aspects should be considered. Firstly, improving photosynthesis efficiency is crucial, as increased sugar synthesis in leaves is beneficial. Secondly, focusing on sugar translocation from leaf to root, which encompasses sucrose export from mesophyll cells, loading into sieve tube complexes aboveground, and unloading and import into root parenchymal cell underground [38]. Thirdly, considering the storage of sucrose in the vacuoles of root parenchymal cells. Lastly, accounting for sucrose consumption in root cells, such as the formation of structural carbohydrates, primarily cell wall components. Thus, genes involved in these aspects may influence sugar distribution. In conclusion, our extensive screening has identified a panel of interesting genes that could provide a foundation for sugar beet breeding.

## Materials and methods

### Plant materials and genome re-sequencing

A diverse worldwide set of 269 sugar beet accessions were collected and then planted at Liangcheng Experimental Station, Inner Mongolia, China (40.502261 N, 112.145304 E) with an altitude of 1459.24 m. The frost-free period is 120 days and the average annual rainfall is 392.37 mm. The concentrations of nitrogen, phosphorus, and potassium in the soil were 1030, 23.09, and 145 mg/kg, respectively. The pH was 7.59 and organic matter content averaged 1.80%. There were 269 planting plots in the experimental area of 1178 m^2^ (62 m × 19 m). The size of each plot was 1.2 m × 2.3 m. The plant spacing was 0.2 m, and row spacing was 0.5 m. The spacing between the plots was 0.5 m. The sowing time was 20 May 2019. Plants were harvested with replicates on 20 September 2019.

When we collected this diverse set of 269 sugar beet accession seeds, we obtained their origin, biomass (high or low), and sugar content (high or low) information. Therefore, we categorized accessions into different groups. However, as actual values of biomass and sugar content may vary among different conditions such as water and nutrient status, we set a middle group of which accessions showed a wide range of values.

For genome re-sequencing, DNA was extracted from each accession. Illumina DNA libraries were constructed according to the manufacturer’s instructions and then sequenced on the Illumina HiSeq PE150 platform, generating 150-bp paired-end reads.

### Sequence alignment, variant calling, and annotation

Clean paired-end reads were mapped to the sugar beet reference genome (version RefBeet-1.2.2) using BWA-MEM [39] with default parameters. Then SAMtools was used to view and sort the BAM files. Next, Picard tools (version 1.96) was used with two modules. First, the Sort module was used to sort the alignment by coordinates; second, the MarkDuplicates module was used to mark duplication alignment. Then we performed local realignment using GATK software [40] (version 3.7.0) with the RealignerTargetCreator and IndelRealigner modules. SNPs were called by the HaplotypeCaller module in GATK. Finally, all vcf files were merged into one vcf file. GATK SelectVariants was used to split SNPs and indels into two files. Hard filtering was applied to the raw variant set using GATK. SNPs were filtered with the following parameters: QD < 2.0 || QUAL < 30.0 || SOR > 3.0|| MQ < 40.0 || FS > 60.0 || MQRankSum < −12.5 || ReadPosRankSum < −8.0. Indels were filtered with the following parameters: QD < 2.0 || QUAL <30.0|| FS > 200.0 || ReadPosRankSum < −20.0. All identified SNPs and indels were further annotated with ANNOVAR software [41] and were divided into groupings of variations occurring in intergenic regions, coding sequences, and introns, on the basis of reference genome annotation information.

### Phylogenetic and population structure analysis

We used a subset of 4 436 506 SNP (SNPs with no missing genotypes and MAF >0.1) in 207 sugar beet accessions to construct the phylogenetic tree using PHYLIP software [42] with 1000 bootstrap replicates. MEGA4 [43] was used to display the phylogenetic tree. We also used this subset of SNPs to conduct PCA using GCTA software [44]. The nucleotide diversity π (—site-pi) and *F*_ST_ (—weir-fst-pop) were calculated using VCFtools [45].

### Linkage disequilibrium analysis

For calculating LD, SNPs with MAF <0.05 and having missing data were removed from LD analysis, resulting in 4.39 million SNPs left. PopLDdecay (v.3.41) software [46] was used to calculate LD values for all accessions. LD decay estimation for SNP (MAF ≥0.05) was measured by pairwise correlation coefficient (*r*^2^) for each SNP pair.

### Phenotype collection

The taproot was harvested 140 days after sowing, then the leaves were cut horizontally; the soil and fine root hairs on the taproot surface were removed, and then an electronic scale was used to measure the weight, which was called biomass. The soluble sugar content was measured by a PAl-1 digital refractometer (ATAGO, Japan, ranging from 0.0 to 53.0%). Ten taproot phenotypic traits were extracted based on the processed three-dimensional point cloud, comprising root length, maximum diameter, convex hull volume, top projection area, compactness, convex index, convex angle, root head ratio, root tail ratio, and root taper index. These traits and calculation formulae were described in detail previously [28].

### Genome-wide association study

In total, 13 319 866 SNPs were generated for 207 accessions. Phenotype data were available for 188 sugar beet accessions. SNPs with an MAF <0.05 were removed. Therefore, 4 436 259 SNPs were used for conducting association mapping. All analyses were done in the R program. We used an EMMA [47] to run the association study. The linear model was:


$$ y=X\mathrm{\beta} +\mathbf{Z} u+e $$

where *y* is phenotype, *X* is a fixed effect including mean and SNP variables; β is a coefficient, **Z** is an incidence matrix mapping each observed phenotype to one of the accessions; *u* is a random effect with $Var(u)={\sigma}_g^2\mathbf{K}$, where **K** is the kinship matrix inferred from SNPs that represents population structure, and *e* is the residual effect.

A *P*-value ≤10^−5^ was used for determining significance.

### RNA-seq analysis

Two accessions (kws2314 and kws1197) were chosen for RNA-seq. Eighteen samples (two accessions, three time points, each with three replicates) were sequenced on the Illumina platform. Then clean reads were aligned to the reference genome (Refbeet 1.2.2) using RSEM [48]. DEGs were identified using the DESeq2 package [49]. All bioinformatics-related statistical analyses were done in R software.

### qRT–PCR of candidate genes

Total RNA was extracted using TRIzol reagent (Invitrogen). The extracted RNA was digested with RNase-free DNase (Takara) and the cDNA was synthesized using M-MLV reverse transcriptase (Takara). The real-time quantitative reverse transcription (qRT)–PCR was performed with Taq Pro Universal SYBR qPCR Master Mix (Vazyme). The candidate genes were analyzed using primers listed in Supplementary Data Table S9. ACTIN was used as the reference gene and relative gene expression was calculated using the 2^−ΔΔCT^ method. The qRT–PCR experiments were performed in triplicate.

## Supplementary Material

Web_Material_uhae230

## Data Availability

The raw sequencing data for 207 accessions were deposited in the NCBI SRA database (PRJNA698015): https://dataview.ncbi.nlm.nih.gov/object/PRJNA698015?reviewer=rhj76bk5bfcu388vaeg6nt5cqs.
