# Supplementary material for: Genome-wide association study identifies the genetic basis of key agronomic traits in 207 sugar beet accessions
Source: Hortic Res. 2024 Aug 12;11(10):uhae230. doi: 10.1093/hr/uhae230 (PMC11481341; doi:10.1093/hr/uhae230)
Supplement: Web_Material_uhae230 [file web_material_uhae230.zip › SupplementaryFigures.docx]

**
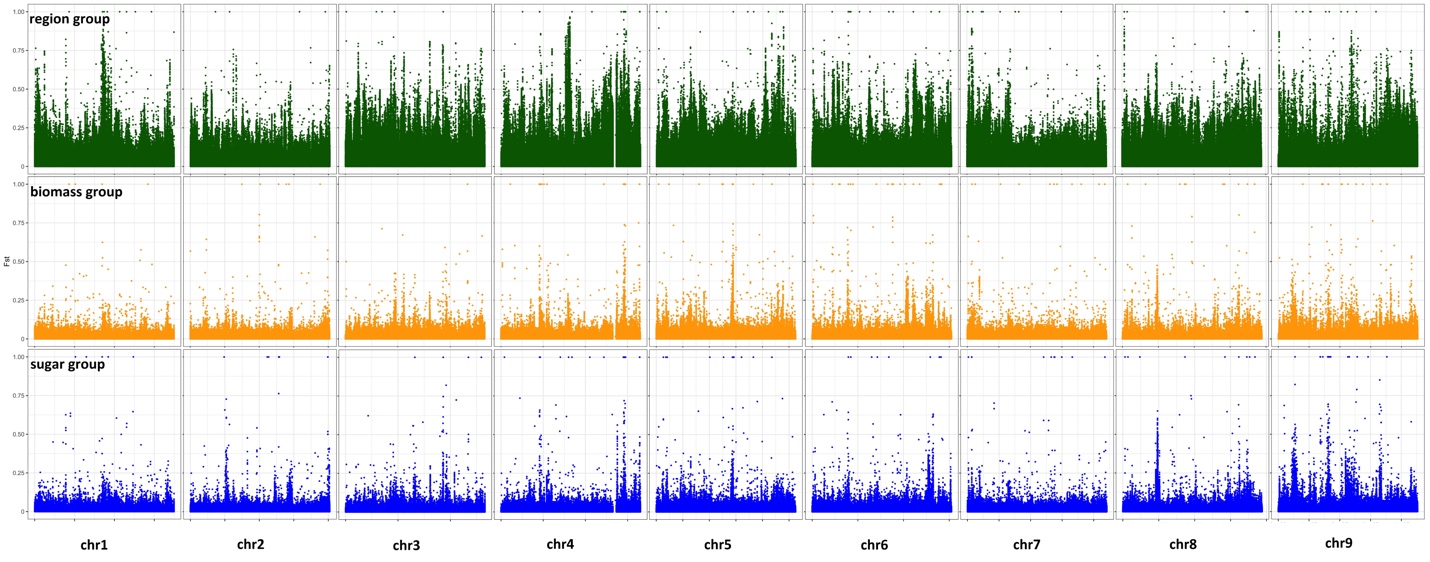
**

**Supplementary Figure 1**. The genome-wide fixation index (*F*_ST_) values based on different groups. The top panel is China and non-China groups, the middle panel is biomass group and the bottom panel is sugar groups.

**
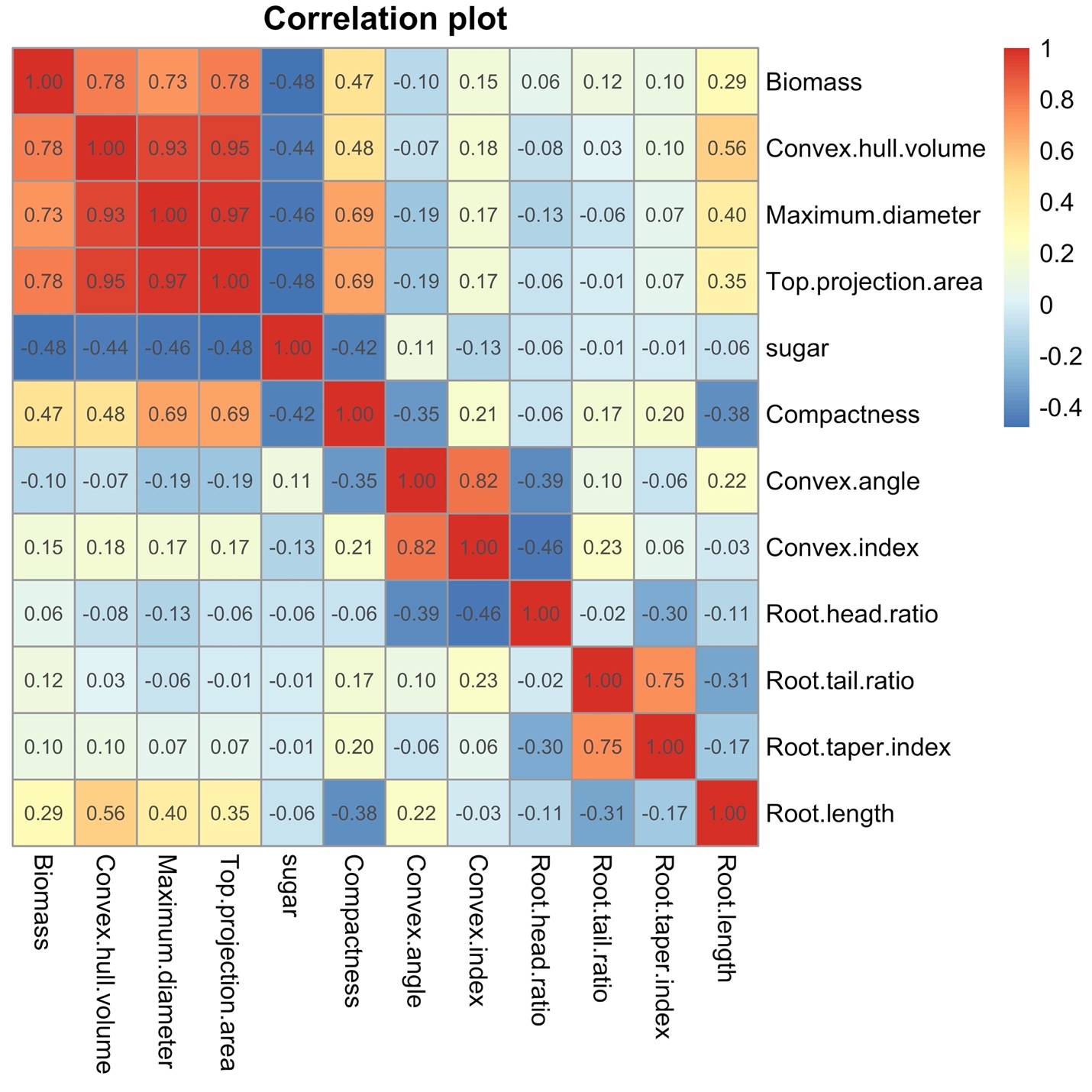
**

**Supplementary Figure 2**. Correlation plot of pairwise correlation analysis for twelve phenotypical traits measured in 188 sugar beet accessions. Postive number indicates a positive correlation between two traits, negative number indicates a negative correlation between two traits.

**
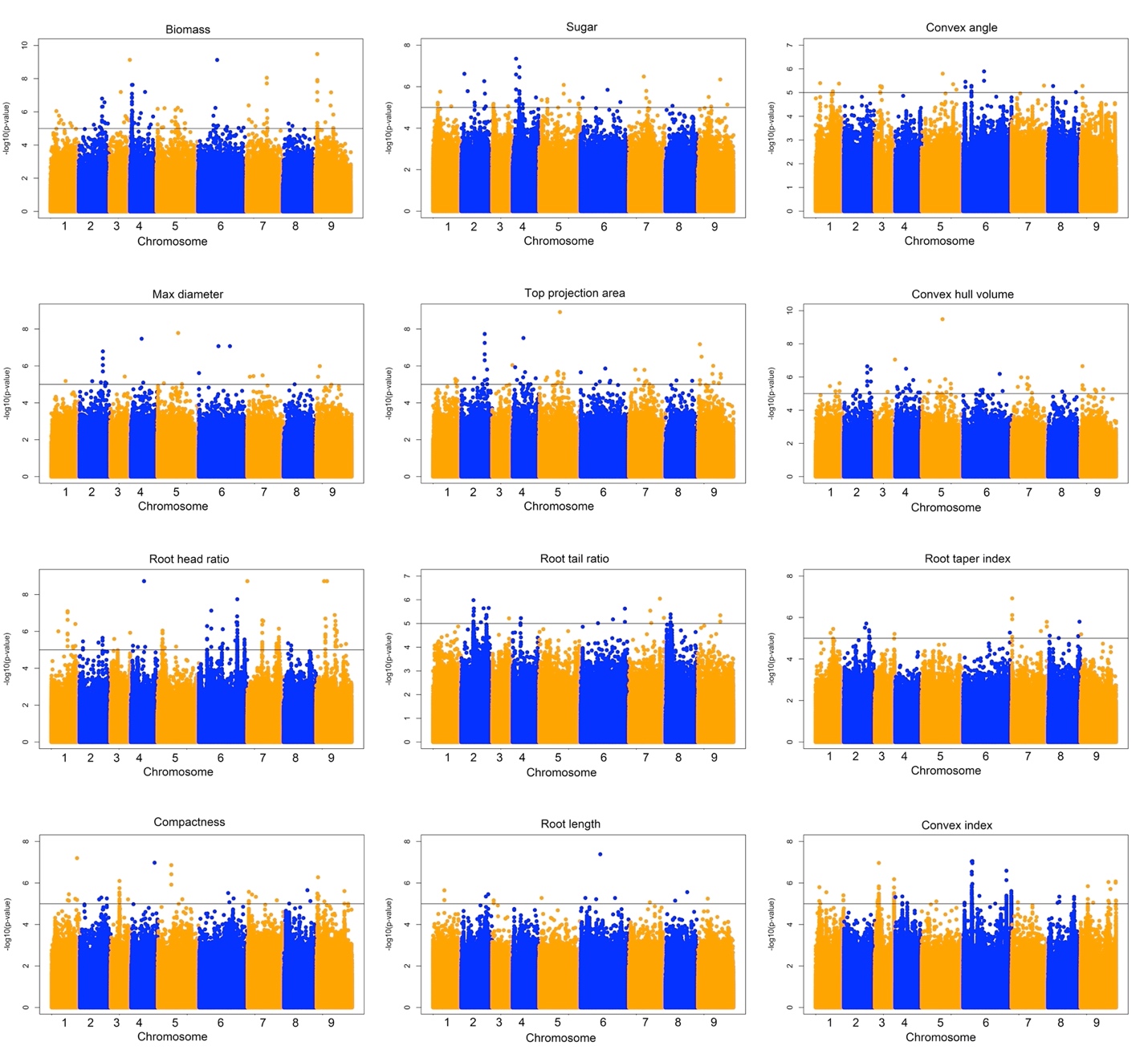
**

**Supplementary Figure 3**. Manhattan plot of genome-wide association study for 12 phenotypical traits in 188 sugar beet accessions including biomass, soluble sugar content and ten taproot traits (convex angle, max diameter, top projection area, convex hull volume, root head ratios, root tail ratio, root taper index, compactness, root length and convex index). The significant threshold was set as *P*-value ≤ 10^-5^.


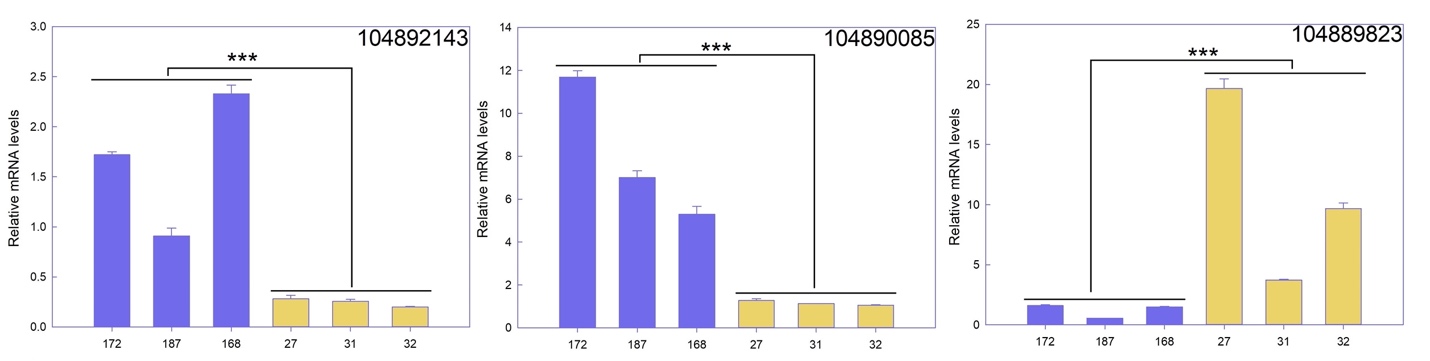


**Supplementary Figure 4**. Expression analysis (qRT-PCR) of three genes that related to biomass synthesis or sugar accumulation. These genes are UDP-glucose 4-epimerase (ID: 104892143), hydroxyproline o-arabinosyltransferase (ID: 104890085) and probable pectinesterase (ID: 104889823). Blue bars are sugar beet accessions from high biomass group, yellow bars are accessions from high sugar accessions. Data are representative of three independent experiments (mean ± SD in panel). Significance level was indicated as: *P*-value < 0.05 as *, *P*-value < 0.01 as ** and *P*-value < 0.001 as ***.


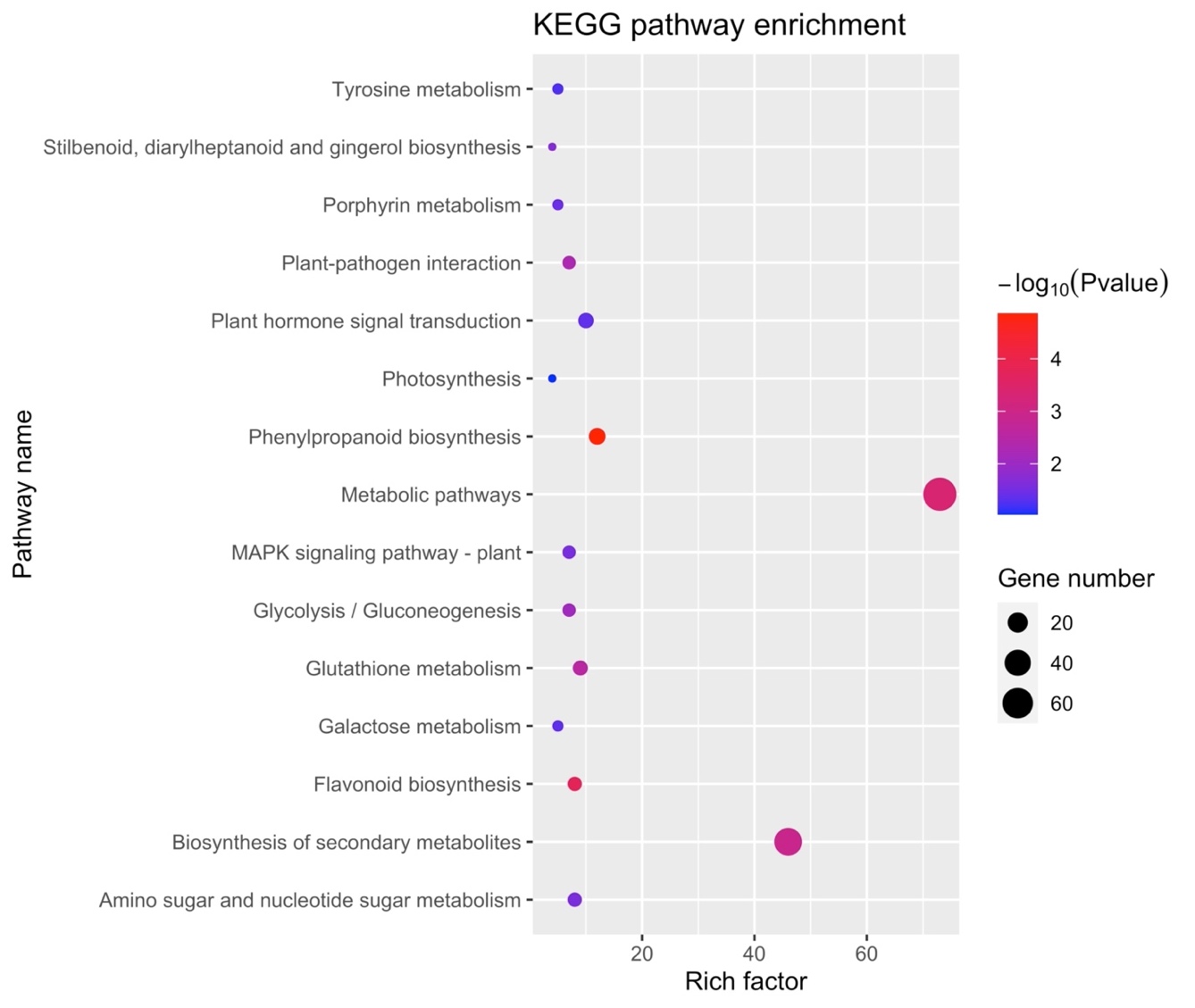


**Supplementary Figure 5**. The Bubble gram of significant KEGG pathways in RNA-seq analysis. The color indicates the significance level, the size of the bubble indicates the number of genes in that pathway.
